# TMEM16A as a potential treatment target for head and neck cancer

**DOI:** 10.1186/s13046-022-02405-2

**Published:** 2022-06-07

**Authors:** Kohei Okuyama, Souichi Yanamoto

**Affiliations:** 1 Department of Periodontics and Oral Medicine, University of Michigan School of Dentistry, Ann Arbor, MI 48109 USA; 2 University of Michigan Rogel Cancer Center, Ann Arbor, MI 48109 USA; 3 Department of Oral and Maxillofacial Surgery, Graduate School of Medical and Dental Sciences, Tokyo Medical and Dental University, Tokyo, 113-8510 Japan; 4 Department of Oral Oncology, Graduate School of Biomedical and Health Sciences, Hiroshima University, Hiroshima, 734-8553 Japan

**Keywords:** TMEM16A, Ca^2+^-activated Cl^−^ channel, TGF-β, EGFR, PD-L1, NF-kB, STAT3, HPV, Proliferation, Metastasis, Head and neck squamous cell carcinoma

## Abstract

Transmembrane protein 16A (TMEM16A) forms a plasma membrane-localized Ca^2+^-activated Cl- channel. Its gene has been mapped to an area on chromosome 11q13, which is amplified in head and neck squamous cell carcinoma (HNSCC). In HNSCC, TMEM16A overexpression is associated with not only high tumor grade, metastasis, low survival, and poor prognosis, but also deterioration of clinical outcomes following platinum-based chemotherapy. Recent study revealed the interaction between TMEM16A and transforming growth factor-β (TGF-β) has an indirect crosstalk in clarifying the mechanism of TMEM16A-induced epithelial-mesenchymal transition. Moreover, human papillomavirus (HPV) infection can modulate TMEM16A expression along with epidermal growth factor receptor (EGFR), whose phosphorylation has been reported as a potential co-biomarker of HPV-positive cancers. Considering that EGFR forms a functional complex with TMEM16A and is a co-biomarker of HPV, there may be crosstalk between TMEM16A expression and HPV-induced HNSCC. EGFR activation can induce programmed death ligand 1 (PD-L1) synthesis via activation of the nuclear factor kappa B pathway and JAK/STAT3 pathway. Here, we describe an interplay among EGFR, PD-L1, and TMEM16A. Combination therapy using TMEM16A and PD-L1 inhibitors may improve the survival rate of HNSCC patients, especially those resistant to anti-EGFR inhibitor treatment. To the best of our knowledge, this is the first review to propose a biological validation that combines immune checkpoint inhibition with TMEM16A inhibition.

## Introduction

**Fig. 1:**
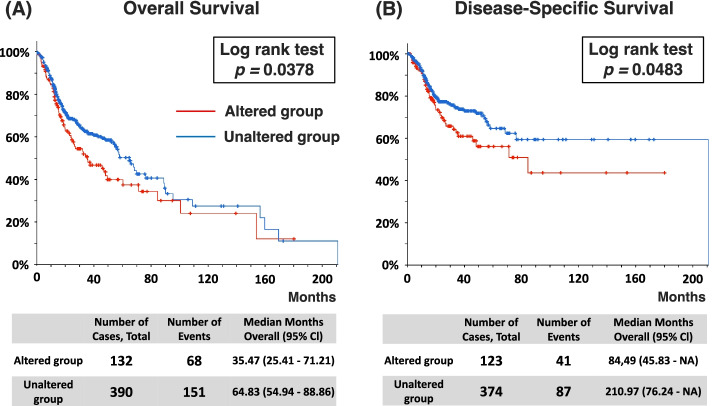
The Cancer Genome Atlas database (http://cancergenome.nih.gov/) showed that TMEM16A-altered head and neck cancer was significantly correlated with shorter (**A**) overall (*p* = 0.0378) and (**B**) disease-specific survival rates (*p* = 0.0483) in patients who underwent surgery

Calcium-activated chloride channels (CaCCs) are abundant in nearly every cell type, where they perform various functions [1]. It is now well-accepted that transmembrane protein 16A (TMEM16A), also known as Anoctamin-1, DOG1, ORAOV2, and TAOS2, forms the localized plasma membrane of the CaCC [2–4]. TMEM16A has been mapped to an area on chromosome 11 (11q13), which is often magnified in cancer cells [5]. Apart from glands, CaCCs have long been known to be present primarily in proliferating cells in culture and various types of cancer cells [1, 6, 7]. TMEM16A is a proven significant and reliable tumor marker, particularly for gastrointestinal stromal tumors (GISTs) and head and neck squamous cell carcinoma (HNSCC) [8, 9]. Duvvuri et al. reported that TMEM16A is substantially amplified and highly expressed in 85% of HNSCC patients and correlates with decreased patient survival [10]. According to the data obtained from The Cancer Genome Atlas (TCGA) database (http://cancergenome.nih.gov/), the overall and disease-specific survival rates of the TMEM16A-altered HNSCC patient group were significantly lower than those of the unaltered group (Fig. 1). Thus, TMEM16A has tumor-specific functions and supports cell proliferation and possible development of malignancy in any cancer cell type [11, 12].

Based on the nature of TMEM16A, many biological experiments have been conducted, and TMEM16A is now considered an excellent biomarker for certain cancer types. On the other hand, Qu et al. insisted that TMEM16A functions in cell growth as an oncogene; its overexpression is not the final step of tumorigenesis as it is involved in the initiation of tumorigenesis, whereas its overexpression is not associated with its amplification [13]. The role of TMEM16A as an oncogene has been well-investigated, however, how TMEM16A interacts with the other biomarkers and related pathways is not fully understood.

HNSCC is an aggressive malignancy with high morbidity and mortality rates. Worldwide, HNSCCs are responsible for over 550,000 new cases and over 380,000 deaths annually [14]. The main risk factors of HNSCCs are long-term tobacco use, alcohol consumption, and infection with high-risk types of human papillomavirus (HPV) [15]. Despite advances in diagnosis and improvements in imaging modalities in recent decades, the survival of patients with HNSCC has remained unchanged owing to the high recurrence rate and high risk of cervical lymph node or distant metastasis [16, 17]. The current standard treatment for HNSCC in most patients is surgery, and postoperative concurrent chemoradiotherapy using platinum-based agents is a widely accepted standard of treatment for patients with a high risk of recurrence, as determined by surgical pathological findings [18, 19]. However, treatments such as radio- and chemotherapy can cause toxicity to other organs that can lead to a reduction in the quality of life. Moreover, therapeutic strategies, particularly for patients with locoregional recurrent or distant metastatic HNSCC, remain limited. Therefore, there is a constant need for improved therapeutic strategies. The most promising option remains targeted therapy [20, 21]. Many CaCC inhibitors have been reported to date. Each substance has been identified with a target organ/structure; however, their practicality for daily clinical use is far from realistic [22].

HPV infection is now one of the prognostic factors in HNSCC, indicating a favorable prognosis [23, 24]. Moreover, this infection can contribute to several functional, genetic, and metabolic changes [25–27], including immuno-escape of HNSCC [28], and can be considered a potential effector for cancer cell metabolism. Some literatures reported the biological and cellular metabolic relationship between the expression of TMEM16A and HPV infection.

A surprising range of biological and molecular functions associated with TMEM16A have emerged from the literature, suggesting that TMEM16A plays a multifaceted role in cancer. In this review, we discuss the current understanding of the molecular dynamics of TMEM16A in cancer, summarise evidence of the biological and molecular functions of TMEM16A, and discuss a potential combination treatment for HNSCC based on TMEM16A inhibition.

### Is TMEM16A really a promising biomarker?

Overexpression of TMEM16A is widely thought to promote cancer cell proliferation and migration. Duvvuri et al. reported that TMEM16A overexpression in HNSCC significantly promoted anchorage-independent growth in vitro, and the loss of TMEM16A resulted in the inhibition of tumor growth both in vitro and in vivo [10]. Ayoub et al. found TMEM16A amplification and expression in HNSCC, accompanied by a high propensity for distant metastasis [29]. In esophageal SCC (ESCC), Shi et al. reported that TMEM16A mRNA expression and protein overexpression were associated with lymph node metastasis (LNM) and advanced clinical stage [30]. LNM is also more common in HNSCC patients with TMEM16A overexpression than in those without [9]. The role of TMEM16A as a prognosticator has been characterized in other organ cancers. Bae et al. found that TMEM16A overexpression was an independent indicator of poor prognosis of shorter overall survival and relapse-free survival of breast cancer patients [31]. A detailed study on the reaction of tamoxifen for breast cancer treatment reported that high TMEM16A expression correlated with decreased survival in patients not treated with tamoxifen and with increased survival in tamoxifen-treated patients [32]. Wu et al. also reported that TMEM16A overexpression is associated with good prognosis in progesterone-positive or HER2-negative breast cancer patients following tamoxifen treatment, especially in patients with low Ki67 expression [33]. Therefore, TMEM16A can serve as a biomarker for clinical outcomes and breast cancer therapeutic responses to tamoxifen [5]. In colorectal cancer (CRC), Li et al. reported that TMEM16A mRNA expression could be a novel predictive marker of LNM, and TMEM16A expression may be an important regulator of tumor proliferation and metastasis [34]. Moreover, Liu et al. (2015) found that high expression levels of TMEM16A were associated with LNM, higher disease grade, and poor prognosis of gastric cancer (GC) [35], whereas Liu et al. (2012) confirmed a positive correlation between TMEM16A expression and tumor grade in prostate cancer [36]. Atala et al. reported that TMEM16A knockdown decreased the proliferation of prostate cancer cells and suppressed xenograft tumor growth in vivo [37]. Britschgi et al. obtained similar results showing that TMEM16A is critical for cell survival and proliferation in 11q13-amplified breast cancer, HNSCC, and ESCC cells [38]. The above data show that overexpression of TMEM16A correlates with high tumor grade, low survival, and poor prognosis. On the other hand, mutations such as S741T increased TMEM16A calcium sensitivity and channel activity, and rendered TMEM16A calcium gating voltage-independent, whereas mutants like L759Q diminished TMEM16A-dependent chloride conductance without affecting TMEM16A expression level or localization on the membrane. One of the key findings is that the S741T mutation could be functional even at very low intracellular calcium levels suggesting that TMEM16A has the capacity to function biochemically in the absence of high calcium in the cytoplasm [39, 40]. It could be that in a tumor cell setting TMEM16A can function in the absence of calcium to drive downstream signaling, with or without chloride channel activity. These dynamics of TMEM16A no longer show a simple ion-channel but a tumor driver, suggesting its potential use as a promising tumor biomarker.

### TMEM16A downstream pathways and crosslink to other pathways

TMEM16A-induced cancer cell proliferation and tumor growth were accompanied by an increase in the induction of extracellular signal-regulated kinase (ERK)1/2 and cyclin D1 [10]. Sui et al. reported that TMEM16A small-interfering RNA decreased the in vitro proliferation of CRC cells by inhibiting the expression of ERK1/2 [41]. It is now well-known that the rat sarcoma (RAS)- rapidly accelerated fibrosarcoma (RAF)-Mitogen-activated protein kinase (MAPK) signaling pathway can activate ERK1/2. Mutations in Kirsten rat sarcoma virus (KRAS), neuroblastoma-RAS (NRAS), and B-RAF have also been demonstrated to be involved in the initiation and progression of CRC [42]. TMEM16A is also overexpressed in hepatocellular carcinoma, and that the inhibition of TMEM16A suppressed MAPK and tumor growth [43]. In addition, it is generally known that mutant p53 overexpression is related to tumor metastasis, recurrence, and poor prognosis. Multivariate statistical analysis by Sui et al. showed that TMEM16A protein expression positively correlated with KRAS mutation status and negatively correlated with mutant p53 protein expression in CRC. Thus, their data suggest that TMEM16A may play a dual role in tumor formation and metastasis by interacting with mutated KRAS and mutant p53 proteins in CRC tissues [41]. Because HNSCC carries a high mutation rate of p53 [44], studying how its gene mutations interact with TMEM16A expression could be a promising direction for future research.

Epidermal growth factor receptor (EGFR) family members (EGFR1, HER2, EGFR3, and EGFR4) can activate many signaling pathways including phosphatidylinositol 3-kinases (PI3K)/PTEN/AKT/Mammalian/mechanistic Target of Rapamycin (mTORC1)/glycogen synthase kinase-3 (GSK-3), Ras/Raf/MEK/ERK, Janus kinase (JAK)/signal transducer and activator of transcription (STAT), and c-Jun-NH2-terminal kinase (JNK) [45, 46]. TMEM16A has been suggested to regulate EGFR signaling. Bill et al. reported that TMEM16A and EGFR form a functional complex that regulates HNSCC cell proliferation. EGFR signaling increases TMEM16A protein levels, and knockdown of TMEM16A reduces EGFR protein levels without affecting EGFR mRNA levels [47]. Moreover, EGF promotes TMEM16A expression in breast cancer cells through the EGFR-STAT3 pathway [32]. STAT3 signaling also plays an important role in programmed cell death-ligand 1 (PD-L1) upregulation and the antitumor immune response of HNSCC [48]. Recently, Luo et al. reported a novel mechanism underlying TMEM16A-mediated breast cancer metastasis; increased TMEM16A channel activities activate EGFR/STAT3/ROCK1 signaling, and ROCK1 activation by RhoA increased TMEM16A channel activities via moesin phosphorylation at T558. TMEM16A and ROCK1/moesin signaling cooperatively promotes breast cancer metastasis [49]. These data suggest that there is a promising link between TMEM16A and EGFR, which regulates the proliferation and metastasis of cancer cells.

TMEM16A expression may be regulated at the translational level by several microRNAs (miRs), such as miR-9, miR-132, and miR-144, as previously reported [50–52]. MiRs can also regulate TMEM16A function. MiR-132 and miR-381 bind to the 3′ untranslated region of TMEM16A mRNA, resulting in TMEM16A downregulation in patients with CRC [52] and GC [53]. Mokutani et al. revealed that TMEM16A was a direct target of miR-132 and its overexpression was inversely associated with the downregulation of miR-132 in CRC, and correlated with poor clinical outcomes in patients with CRC [52]. TMEM16A protein, then, can interact with other molecular targets including ERK1/2, AKT, calmodulin kinase II (CaMKII), and EGFR at the post-translational level [5, 38, 54]. To investigate the unknown mechanisms underlying TMEM16A-related miRs, regulation of cancer progression and metastasis, are required to clarify how those miRs function as messengers among various pathways.

Transforming growth factor-β (TGF-β) plays a key role in controlling embryogenic development, inflammation, and tissue repair, as well as in maintaining adult tissue homeostasis. TGF-β elicits a broad range of context-dependent cellular responses, and consequently, alterations in TGF-β signaling have been implicated in many diseases, including cancer. It is well-known that TGF-β acts as a tumor suppressor during the early phase of cancer progression and as a tumor promotor in advanced stage. During the early stages of tumorigenesis, TGF-β acts as a tumor suppressor by inducing cytostasis and the apoptosis of normal and premalignant cells. However, at advanced stages, when cancer cells have acquired oncogenic mutations and/or have lost tumor suppressor gene function, cells are resistant to TGF-β-induced growth arrest, and TGF-β functions as a tumor promotor by stimulating tumor cells to undergo epithelial-mesenchymal transition (EMT) [55]. Thus, the expression of TGF-β signaling is an important factor that should be considered in the tumor metastasis step. TGF-β is found to be a potent secreted cytokine that drives cancer progression, not only through its immunosuppressive and proangiogenic roles, but also more importantly as a potent inducer of EMT by regulating E-cadherin expression [56]. The activation of TGF-β receptor complex recruit and induce many signaling proteins such as protein and lipid kinases, scaffolding proteins and small GTPases, whereas some of these proteins become directly phosphorylated by the TGF-β receptors [57]. It is now generally accepted that TGF-β and other members of its family control many fundamental aspects of cellular behaviour, including migration, adhesion, differentiation, and modification of the microenvironment [58]. Actually, TGF-β receptor type-I is known as the family of SMAD proteins, which, upon phosphorylation, oligomerize with SMAD4, accumulate in the nucleus and regulate gene transcription by binding to regulatory sequences in the genome together with other transcription factors and chromatin proteins [59, 60].

**Fig. 2:**
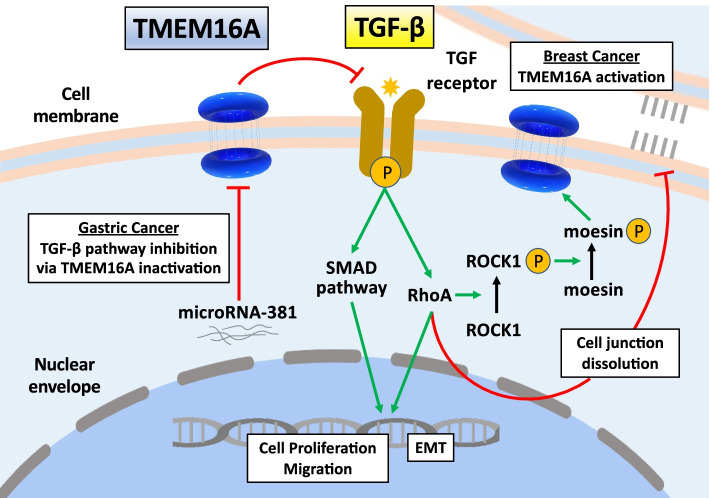
As the canonical signaling, the TGF-β ligand binds to the heterodimeric receptor which regulates the phosphorylation of SMAD2/3 proteins. Then, these proteins form complexes with SMAD4 and initiate transcription. TGF-β downstream pathway can promote tumorigenesis, cell proliferation, and migration by inducing the epithelial to mesenchymal transition (EMT). As one of the non-canonical signaling, TGF-β pathway also can activate RhoA signaling during EMT, and that inhibition of RhoA signaling blocks TGF-β-induced EMT. RhoA is mainly involved in activating stress fibers and cell contraction and its downstream signaling promotes cell junction dissolution. The activation of ROCK1 gene by RhoA increased TMEM16A channel activity owing to the phosphorylation of moesin at T558 in breast cancer cells. On the other hand, MiR-381 function as a tumor suppressor by directly targeting TMEM16A and regulating TGF-β pathway and subsequently EMT process in the development of progression of gastric cancer. The interaction between TMEM16A and TGF-β may have an indirect crosstalk in clarifying the mechanism of TMEM16A-induced EMT. Inhibitory/negative signals are indicated with inhibitory red arrows; Stimulatory/positive signals are indicated with green arrows

Regarding the relationship between TGF-β and TMEM16A, Atala et al. reported that the knockdown of TMEM16A decreased I_Cl,Ca_, impaired TGF-β secretion, reduced E-cadherin expression, and inhibited migration and invasion without affecting the proliferation of prostate cancer cells [37]. In addition, Cao et al. discovered that miR-381 could inhibit the TGF-β signaling pathway and partially downregulate EMT phenotype by targeting TMEM16A [53]. These data suggest that TMEM16A participates in the TGF-β signaling pathway to regulate cell proliferation and migration. Moreover, Liu et al. suggested that TGF-βs might serve as intermediators between TMEM16A and oncogenic signaling such as MAPK. The activation of TMEM16A chloride channel depolarized membrane potential, promoted TGF-β secretion, downregulated E-cadherin expression, and facilitated GC migration and invasion [35]. In addition, one of non-canonical TGF-β pathway can activate RhoA signaling during EMT, and that inhibition of RhoA signaling blocks TGF-β-induced EMT [61, 62]. In this regard, Luo et al. reported ROCK1 increased TMEM16A channel activity via moesin phosphorylation and the increase in TMEM16A channel activities promoted cell migration and invasion. They investigated the activation of ROCK1 gene by RhoA increased TMEM16A channel activity owing to the phosphorylation of moesin at T558 in breast cancer cells [49]. Taken together, the interaction between TMEM16A and TGF-β may have an indirect crosstalk in clarifying the mechanism of TMEM16A-induced EMT. We show the representative sherma on TGF-β pathway and its surroundings related to TMEM16A expression in Fig. 2.

### TMEM16A expression and anti-tumor drug sensitivity

TMEM16A overexpression correlates with improved clinical outcomes following chemotherapy [34]. Vyas et al. reported a strong positive correlation between TMEM16A and ATP7B expressions, which balances the copper level in the body by excreting excess copper into bile and plasma in HNSCC. TMEM16A overexpression and depletion in HNSCC cell lines caused parallel changes in ATP7B expression. A previous study showed that increased oxidative stress in cells overexpressing TMEM16A liberated the chelated copper in the cytoplasm, leading to the transcriptional activation of ATP7B expression, which in turn decreased the efficacy of platinum compounds by promoting their vesicular sequestration [63]. TMEM16A also promotes the sensitivity of other antitumor agents [47, 64], and TMEM16A overexpression could correlate with the deterioration of clinical outcomes by chemotherapy [34]. Platinum-based chemotherapy is the cornerstone treatment for a variety of human malignancies, including HNSCC. Clinically, resistance to cisplatin therapy is a major problem contributing to poor patient outcomes [65]. Godse et al. provided strong evidence that in a portion of cisplatin-resistant malignancies, TMEM16A overexpression may lead to inhibition of apoptosis and subsequent cisplatin resistance. Furthermore, they concluded that this raises the possibility that pharmacologic inhibition of TMEM16A or its relevant downstream effectors, such as ERK1/2, could be strategically used to overcome cisplatin-resistant malignancies in TMEM16A expressing malignancies [66]. Thus, there is a need to construct a platinum-based combination therapy that includes a TMEM16A inhibitor. Moreover, the c-KIT negative GIST that expresses TMEM16A could be imatinib resistant population that could benefit from a TMEM16A inhibitor. There is a need to define the role of TMEM16A in GIST using both c-KIT positive and negative cell lines and xenografts [67].

### TMEM16A expression and HPV infection on HNSCC

HPV + HNSCC is significantly different from HPV − HNSCC in many aspects, such as DNA mutation and mRNA and protein expression. In contrast, HPV − HNSCC is activated by several risk factors and is highly related to TP53 mutations. To date, there have been some reports on the interrelationship between TMEM16A and HPV in HNSCC. Dixit et al. found that TMEM16A was preferentially overexpressed in HPV − HNSCC compared to HPV + HNSCC, and that this overexpression was associated with decreased patient survival. They revealed that promoter hypomethylation contributes to TMEM16A overexpression in HPV − HNSCC [68]. HPV + and HPV − HNSCC are considered two entirely different types of cancer, in part due to their unique molecular landscapes [69]. HPV proteins E6 and E7 downregulate tumor suppressor proteins p53 and Rb, respectively, although their true interactome encompasses a wide variety of cellular targets [70]. Recent work has shown that the E6 and E7 proteins act synergistically to induce HNSCC in a genomic engineered mouse model and that additional targets besides Rb are targeted by E7 [71–73]. E6 and E7 also activate oncogenic signaling pathways, including EGFR and PI3K [74, 75]. This coordinated perturbation of the critical tumor suppressor pathway results in uncontrolled growth and proliferation, although with a mutational landscape that is significantly restricted when compared with HPV − HNSCC [76, 77].

EGFR has been reported as a potential co-biomarker of HPV infection [74, 78]. EGFR is associated with HPV-related prognosis in oropharyngeal cancer [79]. EGFR expression in HPV + cancer may be regulated by multiple factors, including existing complex mechanisms and HPV viral proteins. EGFR signaling can also be affected by the Hippo/YAP pathway, which eventually influences cancer progression in HPV + cervical cancer [80]. Qiu et al. reported that E7 protein downregulates EGFR, which downregulates the phosphorylation of EGFR and inhibits EGFR downstream pathway that in turn induces a better prognosis [81]. Moreover, Cai et al. identified a mechanism of immunoregulation mediated via the STING-TBK1- nuclear factor-kappa B (NF-κB) pathway in HPV + cervical cancer [82]. Taken together, considering that EGFR can establish a functional complex with TMEM16A, there may be an indirect relationship between the expression of TMEM16A and HPV + cancer. Further research could reveal the therapeutic implications of HPV + HNSCC and develop a novel treatment strategy such as establishment of HPV vaccination and proteolysis targeting chimera (PROTAC) for E7 combined with TMEM16A inhibition.

### TMEM16A behavior in HNSCC metastatic regions

#### Cervical lymph node metastasis

Importantly, TMEM16A expression is modulated by tumor progression. Shiwarski et al. reported that TMEM16A overexpression exerts an inhibitory effect on the migration, invasion, and metastasis of HNSCC cells. They showed that primary tumors exhibit a high level of TMEM16A, whereas LNM exhibits a low expression of TMEM16A. This is because promoter hypermethylation results in decreased TMEM16A expression in LNM tissues [83]. In general, TMEM16A expression level can be changed due to cancer heterogeneity, and various subtypes can be observed owing to the role of TMEM16A in cancer cells.

Roepman et al. reported that the gene alteration pattern of LNM in HNSCC was maintained, which emphasizes the importance of the primary tumor gene expression profile for the development and treatment of metastasis [84]. In addition, Sugahara et al. reported a comprehensive physical map of the 11q13 amplification region and identified two core regions separated by a breakpoint. Copy number amplification of CTTN (core 2) and/or TPCN2/MYEOV (core 1) in the 11q13 region is strongly associated with LNM in patients with HNSCC. They concluded that the detailed assessment of selected loci, followed by real-time quantitative PCR, appears to be a valid strategy for identifying genetic markers in regions with complicated alterations, as is the case with 11q13. However, despite TMEM16A belonging to 11q13, no significant expression of TMEM16A as a single marker for cervical LNM was detected [85]. Therefore, TMEM16A expression alone may not be a promising marker for predicting LNM in HNSCC but evaluating core-combination gene status may improve the prediction accuracy.

#### Bone metastasis

According to a previous study, the reported frequency of the bone metastasis of HNSCC is approximately 15–39% of distant metastasis depending on the study population and other complicated factors [86]. Unlike in LNM, gene expression in the tumor microenvironment and functional heterogeneity in the bone metastatic region can be different from their primary form because bone resorption occurs due to the activity of osteoclasts, multinucleated cells derived from the haematopoietic colony forming a unit-granulocyte macrophage/monocyte-macrophage family [87]. When cancer cells arrest in the bone, the bone becomes a storehouse of a variety of cytokines and growth factors, such as TGF-β, and thus provides a fertile environment for cancer cell growth. In breast cancer cells, cell-to-cell interaction between vascular cell adhesion molecule 1-positive bone-tropic cells and very late antigen 4-positive osteoclast precursors activate the survival signal through the PI3K/AKT pathway; osteoclast precursors differentiate into osteoclasts through the receptor activator of NF-κB ligand (RANKL)-RANK signaling to promote osteolysis. When osteolysis due to bone metastasis of cancer is advanced, TGF-β is released from the bone matrix that promotes cell growth [88]. As mentioned, TGF-β signaling pathway is a promising treatment target inhibiting TMEM16A-mediated tumor progression and EMT phenotype [32]. At this time, we could not find a manuscript that demonstrated a difference in the expression patterns of the TMEM16 family members in the bone metastatic region. Since TMEM16A functions as a CaCC, the authors expect that the dynamics of the TMEM16 family in the bone metastatic site can be largely different from the primary cancer or other soft tissue metastatic cancers and extracellular Ca^2+^ population may be considerably high in the bone metastatic microenvironment. We believe that there may be a crosstalk between these factors and TMEM16A in the bone metastatic region to drive tumor progression in the bone metastatic microenvironment.

### Inhibition of TMEM16A

There are at least two ways to explain the relationship between TMEM16A overexpression and tumorigenesis. First, TMEM16A overexpression is a causal factor for tumorigenesis. If TMEM16A functions as an oncogene, it participates in tumorigenesis initiation. Second, the overexpression of TMEM16A may be an end step in tumorigenesis. In either case, inhibition of this channel could interrupt tumorigenesis. In particular, if the factors upstream of TMEM16A transcription could be revealed, inhibition of these factors may prevent the overexpression of TMEM16A and help stop the tumorigenic process that includes multiple progressive pathways, such as the EGFR downstream pathway [89]. From this viewpoint, ROCK1 gene, which was revealed by Luo et al., is an indirect TMEM16A driver that might be a reasonable inhibitor for TMEM16A overexpression [49].

To date, including natural products, quite a few TMEM16A inhibitors have been developed. T16Ainh-A01 reduced TMEM16A expression in SKBR3 breast cancer cells [64], and in AR42J cells [90]. Bill, et al. reported that CaCCinh-A01 promoted loss of TMEM16A proteins via endoplasmic reticulum-associated proteasomal degradation of TMEM16A in HNSCC cell lines [91]. Arctigenin and dehydroandrographolide also decreased the protein expression of TMEM16A in lung adenocarcinoma cells [92] and colorectal cancer cells [93], respectively. Moreover, Wang, et al. found that simvastatin, a widely used cholesterol-lowing drug, suppressed TMEM16A channel activities, and inhibited cell proliferation in OSCC cells via TMEM16A in a mevalonate acid-independent way [94]. In addition, luteolin, a common dietary flavonoid, has various biological effects including anticancer activity against multiple types of human cancer cell lines [95]. Seo, et al. reported that luteolin inhibited cell proliferation and migration of PC-3 cells expressing high levels of TMEM16A more potently than that of TMEM16A-deficient PC-3 cells. Notably, luteolin not only inhibited TMEM16A channel activity, but also strongly decreased protein expression levels of TMEM16A [96]. Miner et al. identified niclosamide as a potent inhibitor of TMEM16A [97]. Niclosamide is an anthelminthic drug that was also shown to inhibit NOTCH signaling [98], a pathway that is well known to participate in tumorigenesis [99]. Niclosamide was also shown to inhibit NF-κB, Wnt/ß-catenin signaling, the IL-6-JAK1-STAT3 pathway, GSK-3 and more [100–108]. Moreover, homoharringtonine was reported as a novel natural product inhibitor of TMEM16A by Guo, et al. [109]. Homoharringtonine inhibited TMEM16A activity in a concentration-dependent manner and cancer cell proliferation by inhibiting protein and DNA syntheses [110]. In breast cancer, homoharringtonine suppresses cell growth and promotes apoptosis by regulating the miR-18a-3p-AKT-mTOR signaling pathway [111]. Zhang X, et al. reported cepharanthine as selective TMEM16A inhibitors with potential for lung adenocarcinoma therapy [112]. In addition, they found some avermectins have limited effects on the proliferation, migration, and apoptosis of LA795 cells. Avermectins could also dramatically inhibited the growth of xenograft tumors in mice [113]. Zhang G, et al. identified benzophenanthridine alkaloids inhibited proliferation, migration, and induced apoptosis of lung adenocarcinoma cells [114]. Shi, et al. reported theaflavin effectively inhibited the proliferation and migration of TMEM16A high-expressing lung adenocarcinoma cells, displaying antitumor potential [115]. Guo, et al. reported silibinin as a novel TMEM16A inhibitor for lung adenocarcinoma which induced apoptosis and downregulation of cyclin D1 [116]. Shi, et al. identified zafirlukast as a novel TMEM16A channel inhibitor with excellent anticancer activity for lung adenocarcinoma LA795 cells and in vivo study [117]. Duo, et al. also reported matrine is an effective and safe TMEM16A inhibitor in vitro and in vivo [118]. Bai, et al. reported administration of nuciferine, a novel TMEM16A inhibitor, significantly enhanced the cancer therapy effect of cisplatin and counteracted the toxicity of high concentrations of cisplatin [119]. Ani9 is reported to inhibit the proliferation of TMEM16A overexpressed malignant glioma cell [120]. Since these inhibitors are relatively novel, and in some of them, a mechanism of TMEM16A inhibition is unclear, more basic experiments will be needed (Table 1). Other, there are also lots of TMEM16A inhibitors which are not still fully investigated the anti-tumor effect; MONNA [121], AACT [122], digallic acid [123], evodiamine, rutecarpine [124], purpactin A [125], nimodipine [126], and more. Actually, blocking TMEM16A may be difficult when clarifying the intended target because inhibition of TMEM16A can suppress multiple pathways. Therefore, to find a combined anti-tumor agent with a TMEM16A inhibitor, more studies are needed before clinical trials are conducted.

**Table 1:** Representative TMEM16A inhibitors for cancer

Name	Target	Cancer types	Reported year	References
T16Ainh-A01	NF-kB, EGFR signaling	Breast, HNSCC, Pancrea	2014-	[64] [90]
CaCCinh-A01	ER-associated proteasomal degradation	HNSCC	2014	[91]
Arctigenin	MAPK pathway	Lung adenocarcinoma	2020	[92]
Dehydroandrographolide	Directly inhibit TMEM16A	Colorectal	2015	[93]
Simvastatin	Mevalonate acid-dependent pathway	OSCC	2021	[94]
Luteolin	PI3K/AKT, NF-kB, XIPA, p53	Prostate	2008-	[95] [96]
Niclosamide	NOTCH signaling, NF-kB, Wnt/beta-Catenin signaling, IL-6-JAK1-STAT3 pathway, GSK-3, other	Leukemia, OSCC, Ovarian, Hepatocellular carcinoma	2009-	[97–108]
Homoharrimgtonine	MiR-18a-3p-AKT-mTOR pathway	Lung, Breast, Leukemia	2019-	[109–111]
Cepharanthine	Detailes unknown	Lung adenocarcinoma	2021	[112]
Avermectin	Endogenous TMEM16A-mediated currents	Lung adenocarcinoma	2020	[113]
Benzophenanthridine alkaloid	Endogenous TMEM16A currents	Lung adenocarcinoma	2020	[114]
Theaflavin	Directly inhibit TMEM16A	Lung adenocarcinoma	2021	[115]
Silibinin	Apoptosis, Cyclin D1	Lung adenocarcinoma	2021	[116]
Zafirlukast	Directly inhibit TMEM16A	Lung adenocarcinoma	2022	[117]
Matrine	Directly inhibit TMEM16A and its currents	Lung adenocarcinoma	2018	[118]
Nuciferine	Enhance anti-cancer effect of cisplatin	Lung adenocarcinoma	2022	[119]
Ani9	Directly inhibit TMEM16A	Malignant glioma	2016	[120]

Surprisingly, TMEM16A is also known to be activated by noxious heat over 44 °C [127]. Further research that focuses on the relationship between heat and TMEM16 family diversity, including the relationship with the release of heat shock proteins that are associated with tumor-specific antigens from heat-stressed or dying tumor cells that are phagocytised by antigen-presenting cells are needed [128]. High temperature-focused research may reveal another viewpoint of hyperthermia treatment combined with TMEM16A inhibitor.

### Radiotherapy combined with TMEM16A inhibitor

Radiotherapy is one of a promising treatment strategy for cancer. In HNSCC, combination of high-dose cisplatin administration with radiotherapy is recommended as a standard of care for the postoperative HNSCC patients who are at intermediate/high risk of recurrence [129]. In various cancer, the development of effective radiosensitizers can improve the survival rates. At this time, we cannot detect papers which analyzed TMEM16A as a potential radiosensitizer. It is well-known that TGF-β promotes radioresistance by inducing EMT, cancer stem cells and cancer-associated fibroblasts, suppresses the immune system and facilitates cancer resistance [130]. Moreover, as mentioned, miR-381 functions as a tumor suppressor by directly targeting TMEM16A and regulating TGF-β pathway and subsequently EMT process in the progression of GC [53]. Thus, the inhibition of TMEM16A may gain radiosensitivity via inhibition of TGF-β downstream pathway. On the other hand, hypoxia also enhances the radioresistance, and most advanced solid cancers are hypoxic [131, 132]. There is also no report about the relationship between hypoxia and TMEM16A expression in cancer, but in normal epithelia, such as in cultured sinonasal epithelial layers, long-term hypoxia can enhance TMEM16A expression [133]. Taken together, TMEM16A inhibitor is a candidate for the potential radiosensitizer. The analysis for the relationship between hypoxia and TMEM16A in cancer and the development of reoxygenation methods will be needed in the future experiments.

### TMEM16A and Immune-checkpoint inhibition

Several clinical trials using immune-checkpoint inhibitors have been conducted, and their results have led to the development of pembrolizumab as a first-line treatment for recurrent/metastatic HNSCC [134, 135]. However, PD-L1 expression alone did not completely capture the sensitivity of HNSCCs to PD-1 inhibitors. Furthermore, most patients who initially respond to PD-1 inhibitors eventually develop acquired resistance to immunotherapy through mechanisms that have not yet been completely understood [136].

To date, there are no reports highlighting the use of combination therapy with TMEM16A inhibitors and immune-checkpoint inhibitors. Several studies reported that high PD-L1 expression was associated with the presence of EGFR mutations in non-small cell lung cancer and was an independent negative prognostic factor for this disease [137–139]. Moreover, Xu et al. reported that HPV + HNSCC patients display improved outcomes with PD-1/PD-L1 axis blockade compared to HPV − HNSCC patients. These improved outcomes are likely driven to a greater extent by anti-PD-L1 inhibitors [140]. Sirianant et al. indicated that HPV infections and cancer caused by mutations in TMC8 are related to upregulated Zn^2+^/Ca^2+^ signaling and activation of TMEM16A in HNSCC [141]. Moreover, according to Finegersh et al., three CpG islands near the TMEM16A transcriptional start site correlate with TMEM16A expression, including two that positively correlate with TMEM16A expression. They found that positively correlated CpGs drive TMEM16A expression, revealing this to likely be a fundamental mechanism for regulating TMEM16A in HNSCC. Moreover, because HPV E7-, but not E6-transfected cancer cells led to hypermethylation of a positively correlated CpG island without a change in TMEM16A expression, they concluded that this hypermethylation caused only by E7 was not sufficient to increase TMEM16A mRNA levels that showed the clinical relevance of both negatively and positively correlated CpG expression levels [142]. Taken together, HPV infection can indirectly stimulate TMEM16A expression and function.

Overall, there can be an indirect relationship among the expression of TMEM16A, HPV infection, and PD-L1 and EGFR expression. Here, we show the downstream pathways involved in PD-L1 expression. Both HPV infection and EGFR downstream can affect the NF-κB pathway, a central player in inflammation and immunity, which then forms the p65 and p50 complex. Its transcript COPS5 inhibits PD-L1 ubiquitination; thus, more PD-L1 can be synthesized. Moreover, Antonangeli et al. reported that the upregulation of PD-L1 in cancer cells is controlled via NF-κB downstream of several signals, including oncogene- and cell stress-induced pathways, inflammatory cytokines, and chemotherapeutic drugs [143]. NF-κB has emerged as a key positive regulator of PD-L1 expression. Moreover, some groups have reported an indirect link between EGFR and PD-L1 expression via NF-κB in non-small cell lung cancer [144, 145]. In fact, even in HNSCC, PD-L1 is significantly elevated in cancer models of acquired cetuximab resistance [146]. In addition, Chen et al. reported that STAT3 also has been shown to be key transcriptional regulators of PD-L1 in cancer [147].

TMEM16A can also promote cancer progression by activating EGFR and CAMK signaling. Knockdown of TMEM16A contributes to a reduction in AKT, ERK1/2, and v-Src sarcoma viral oncogene homologue (SRC) phosphorylation levels due to decreased autocrine EGFR ligand secretion, reduced EGFR signaling, and decreased cell viability in breast [38] and ovarian cancer cells [148]. Furthermore, TMEM16A inhibition of its chloride-channel activity reduced CaMKII signaling, which subsequently attenuated AKT, SRC, and ERK1/2 activation in breast cancer [38]. Bill et al. also discovered that TMEM16A and EGFR form a functional complex that jointly regulates HNSCC cell proliferation: EGFR signaling increases TMEM16A protein levels, whereas knockdown of TMEM16A reduces EGFR protein levels [47]. MAPK signaling pathway is an evolutionarily conserved kinase module that controls fundamental cellular processes such as growth, proliferation, differentiation, migration, and apoptosis [149]. Almaça et al. reported that TMEM16A was activated by ATP through an increase in intracellular Ca^2+^ and a Ca^2+^-independent mechanism involving ERK1/2 [150]. These data indicated that the expression of TMEM16A is also functionally linked to the MAPK pathway.

**Fig. 3:**
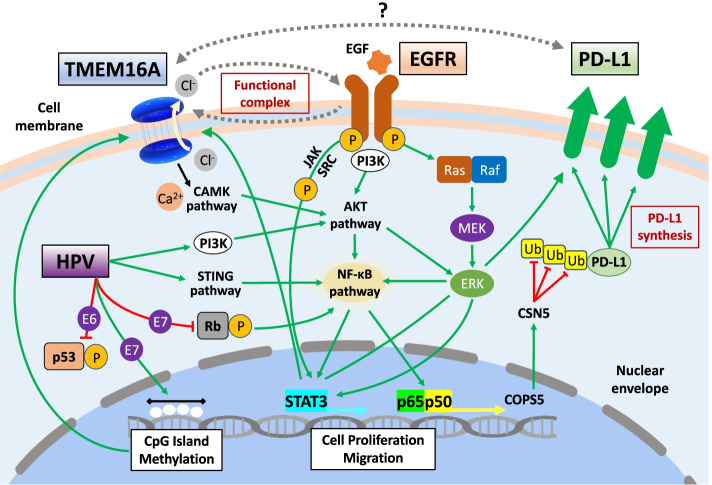
Schematic outline of crosstalk among TMEM16A, EGFR, and PD-L1 in cancer cells. TMEM16A and EGFR interlock and work as a functional complex. The downstream pathway of EGFR in relation to TMEM16A: JAK/STAT, NF-κB, and PI3K/AKT pathways function in the proliferation of cancer cells. As illustrated, the auto-phosphorylation of PI3K leads to the activation of AKT. Downstream of TMEM16A, Ca2 + -mediated activation of CAMK pathway also activates AKT and subsequently activates NF-κB pathway. In HPV infected cells, E7 protein degradates phospho-Rb, activates NF-κB pathway. Moreover, STING-TBK1-NF-κB pathway mediates immunoregulation in HPV + cancer. Both pathways, then, collaborate to NF-κB pathway. E7 protein also affected CpG island methylation, and this positively correlates with TMEM16A expression on cell epithelia. Moreover, EGF promotes TMEM16A expression in breast cancer cells through the EGFR-STAT3 pathway. STAT3 signaling in HNSCC also plays an important role in PD-L1 upregulation. The heterodimer p65/p50 is released and migrates to the nucleus where it undergoes a series of posttranslational modifications including phosphorylation, acetylation, and methylation and binds to specific κB sites and activates NF-κB target genes. In cancer cells, NF-κB activation directly induces expression of the COPS5 gene encoding CSN5, which deubiquitinates and stabilizes PD-L1 protein, suggested increase of TMEM16A-mediated PD-L1 expression may indirectly increase the effect of anti-PD-L1 inhibition, and TMEM16A-mediated driven EGFR pathway may also increase the effect of anti-EGFR inhibitor. Combination therapy using TMEM16A and PD-L1 inhibitors may be a promising strategy to improve the survival rate of head and neck cancer patients especially when tumor gains resistance to anti-EGFR inhibitor treatment. Inhibitory/negative signals are indicated with inhibitory red arrows; Stimulatory/positive signals are indicated with green arrows

Considering the above-mentioned crosstalk between EGFR and TMEM16A, we can describe the indirect interplay among the expressions of EGFR, PD-L1, and TMEM16A (Fig. 3). Combination treatment using a TMEM16A inhibitor and an anti-PD-L1 inhibitor may be a promising strategy. Considering that the co-inhibition of EGFR and TMEM16A had an additive effect on HNSCC cell proliferation, adding PD-L1 inhibition may have a synergistic effect. Moreover, co-targeting of TMEM16A and EGFR could enhance the clinical potential of EGFR-targeted therapy and might delay or prevent resistance to single anti-EGFR inhibitor treatment [47]. Taken together, the authors believe that a combination therapy using anti-TMEM16A and anti-PD-L1 inhibitors can be a promising strategy to improve the survival rate of HNSCC, particularly when the tumor gains resistance to anti-EGFR inhibitor treatment.

This hypothesis may prove useful in the near future. In fact, Katsurahara, et al. reported that ANO9, one of the TMEM family members, promotes cancer growth via STAT3 pathway and regulates PD-L2 expression via interferon (IFN)-related genes in GC. They also found that the knockdown of ANO9 attenuated the PD-L2 expression when IFNα was added in an in vitro analysis. Conversely, PD-L1 increased when IFNα was added [151]. In the case of ANO9, IFNα may play a crucial role for the control of PD-L1 and PD-L2 expression. This study evidently showed the possibility of a crosstalk between the expressions of TMEM family members and PD-L1.

### Prospective

Further research is required to elucidate how the disparity between the intra- and extracellular ion distribution modulates EGFR, PD-L1, and TMEM16A expressions and how it affects the formation and function of the PD-1/PD-L1 axis. Moreover, investigation of the expression levels of EGFR, PD-L1, and TMEM16A in the early-stage HNSCC is necessary to confirm whether the target therapy we suggested in this review can be adopted in such an early disease. In addition, our previous study revealed that prolonged cetuximab treatment induced the inhibition of HNSCC cell migration, resulting in high cell density-related cell stress and persistent cell cycle arrest at G1 phase culminating in autophagy [152]. Hence, we cannot ignore how activated autophagy modulates the intracellular crosstalk among TMEM16A-EGFR-PD-L1-related proteins. Alternatively, targeted protein degradation using a bifunctional PROTAC consisting of a TMEM16A-binding molecule coupled to a degradation inducing moiety could present a promising strategy to target TMEM16A in cancer [40]. In innate immune sensing, further investigations should also focus on how TMEM16A overexpression modulates intratumoral lymphocyte populations and surrounding T lymphocyte recruitment and their functions. Dr Laura Conforti mentioned that Ca^2+^ is in fact a key regulator of the activity of important transcription factors that control transcriptional activity in T lymphocytes. The overall T lymphocyte activation process resides in ion channel proteins controling the Ca^2+^ signaling triggered by antigen presentation that is necessary for T lymphocyte proliferation and cytokine release [153]. Moreover, intratumoral hypoxia and immunity have been correlated with patient outcome in various tumor settings [154], and with EGFR expression [155]. Investigating the relationship between these factors involving treatment resistance and TMEM16A expression is necessary in the future.

## Conclusion

In the present review, we have elaborated on TMEM16A as a promising target in the treatment of HNSCC because it can modulate cell metabolism through interactions with EGFR and PD-L1. This indicates the possibility of combining synergistic therapy with immune-checkpoint inhibitors. To our knowledge, this is the first review to present a validation and mechanism that combines immune-checkpoint inhibitor with TMEM16A inhibition. Considering that the overexpression of TMEM16A is a common feature observed in multiple cancers, suggesting a conserved mechanism for the promotion of carcinogenesis, tumor proliferation, and migration, a crosstalk may exist among these hallmarks in HNSCC.

## Data Availability

Not applicable.

## Abbreviations

CaCC: Ca^2+^-activated Cl^−^ channels
TMEM16A: Transmembrane protein 16A
GISTs: Gastrointestinal stromal tumors
HNSCC: Head and neck squamous cell carcinoma
TCGA: The Cancer Genome Atlas
HPV: Human papillomavirus
LNM: Lymph node metastasis
ESCC: Esophageal squamous cell carcinoma
CRC: Colorectal cancer
GC: Gastric cancer
ERK: Extracellular signal-regulated kinase
MAPK: Mitogen-activated protein kinase
RAS: Rat sarcoma
RAF: Rapidly accelerated fibrosarcoma
EGFR: Epidermal growth factor receptor
PI3K: Phosphatidylinositol 3-kinases
mTORC1: Mammalian/mechanistic target of rapamycin complex-1
GSK-3: Glycogen synthase kinase-3
PD-L1: Programmed cell death-ligand 1
miRs: MicroRNAs
CaMKII: Calmodulin kinase II
TGF-β: Transforming growth factor-β
EMT: Epithelial-mesenchymal transition
PROTAC: Proteolysis targeting chimera
NF-κB: Nuclear factor-kappa B
CpG: Capsid protein gene
STING: Stimulator of interferon genes
STAT: Signal transducer and activator of transcription
JAK: Janus kinase
JNK: C-Jun-NH2-terminal kinase
RANK: Receptor activator of nuclear factor-kappa B
RANKL: Receptor activator of nuclear factor-kappa B ligand
IFN: Interferon
